# Laser Pyrolysis of Imprinted Furan Pattern for the Precise Fabrication of Microsupercapacitor Electrodes

**DOI:** 10.3390/mi11080746

**Published:** 2020-07-30

**Authors:** Jina Jang, Jeong Woo Yeom, Won Kyu Kang, Muhammad Refatul Haq, Xun Lu, Dongjun Shin, Seok-Min Kim, Jung Bin In

**Affiliations:** 1 Department of Mechanical Engineering, Chung-Ang University, 84 Heukseok-ro, Dongjak-gu, Seoul 06974, Korea; jangjina94@cau.ac.kr (J.J.); papillon14@cau.ac.kr (J.W.Y.); zk273rvlb@cau.ac.kr (W.K.K.); refat@cau.ac.kr (M.R.H.); djshin@cau.ac.kr (D.S.); 2 Department of Mechanical Engineering, Yanbian University, Yanji 133002, China; luxun@cau.ac.kr

**Keywords:** laser pyrolysis, imprinting, furan, micropattern, microsupercapacitor

## Abstract

The design or dimension of micro-supercapacitor electrodes is an important factor that determines their performance. In this study, a microsupercapacitor was precisely fabricated on a silicon substrate by irradiating an imprinted furan micropattern with a CO_2_ laser beam under ambient conditions. Since furan is a carbon-abundant polymer, electrically conductive and porous carbon structures were produced by laser-induced pyrolysis. While the pyrolysis of a furan film in a general electric furnace resulted in severe cracks and delamination, the laser pyrolysis method proposed herein yielded porous carbon films without cracks or delamination. Moreover, as the imprinting process already designated the furan area for laser pyrolysis, high-precision patterning was achieved in the subsequent laser pyrolysis step. This two-step process exploited the superior resolution of imprinting for the fabrication of a laser-pyrolyzed carbon micropattern. As a result, the technical limitations of conventional laser direct writing could be overcome. The laser-pyrolyzed carbon structure was employed for microsupercapacitor electrodes. The microsupercapacitor showed a specific capacitance of 0.92 mF/cm^2^ at 1 mA/cm^2^ with a PVA-H_2_SO_4_ gel electrolyte, and retained an up to 88% capacitance after 10,000 charging/discharging cycles.

## 1. Introduction

Laser direct writing is a widely used patterning technique that enables the fabrication of high-resolution micro units without the aid of a photomask. In general, a laser beam is focused on a microscale spot, and a selected area of workpiece is scanned with the beam along a programmed path. This process is often conducted under regular ambient conditions because of the highly instantaneous state change in the spot. Thus, this method has been widely adopted for many applications by both industry and academia [1,2,3,4,5,6,7].

Among its various applications, laser-assisted microscale carbon patterning on polymer surfaces has attracted significant interest in fields related to energy storage devices [8,9,10,11]. Under laser irradiation, the polymer undergoes pyrolysis because of laser-induced heat, yielding electrically conductive and porous carbon structures [9,12,13,14,15,16]. Thus, the laser beam scanning of a polymer film enables microscale carbon patterning, leaving the unaffected polymer area pristine [9,17].

Despite the capability of facile micropatterning, laser direct writing suffers from technical limitations with respect to pattern resolution because of the limited laser beam spot size. Although the resolution can be improved using a tightly focused small beam, the small beam spot would necessitate an increase in the number of beam sweeps for raster scanning. Moreover, laser pyrolysis is mostly photothermal, and the carbonization area widens into the heat-affected zone [13]. As a result, a circular region is formed at the start/end points and at the corners where the beam scanning path changes. In addition, the inertial effect of the laser scanning system can cause the prolonged laser irradiation of deceleration/acceleration regions, resulting in an abnormal temperature rise [18]. High-end laser engravers can mitigate this problem, but the equipment is costly.

The microsupercapacitor is a promising concept to power microelectronics, where microsensors and wireless data transmitters require an energy storage component with a high power density [19,20]. In contrast to conventional sandwich-type supercapacitors, microsupercapacitors have a planar electrode configuration. That is, anode and cathode electrodes of the microsupercapacitor are placed in the same plane; thus, their miniaturization is readily implemented based on micropatterning techniques. The performance of the microsupercapacitor is mainly determined by the electrode material and electrode design. As mentioned above, laser-pyrolyzed carbon can be easily patterned in a microscale based on laser direct writing. Many studies have demonstrated the use of this technique to fabricate microsupercapacitors [21,22,23,24]. The produced carbon exhibited a promising electrochemical performance [25,26,27]. Graphene-like carbon could be also produced by the laser pyrolysis of polymer and referred to as laser-induced graphene [26]. The design or dimensions of a microsupercapacitor are the other important factors that determine its performance. For instance, Pech et al. demonstrated significant effects of different microsupercapacitor electrode dimensions on the specific capacitance, energy density, and equivalent series resistance (ESR) [28]. In this respect, the laser-based fabrication of a high-resolution electrode pattern is crucial especially for the facile on-chip integration of a microsupercapacitor into a microelectronic device [16,19,29,30].

Herein, we propose a laser-induced polymer pyrolysis process to fabricate precise micropatterns of porous carbon. This process involves an imprinting step to produce a precise polymer pattern before laser pyrolysis. At the optimal power and scanning speed, subsequent laser irradiation can efficiently carbonize the polymer pattern, with minimal disturbance to the original imprinted structure. This approach can overcome the abovementioned limitations of laser direct writing patterning. To demonstrate the feasibility of this method, an interdigitated polymer micropattern was imprinted using a polydimethylsiloxane (PDMS) mold and a furan resin with a high carbon content (~40–50%) [31,32]. The imprinted furan pattern was then carbonized by raster scanning with a CO_2_ laser. Finally, the obtained carbon pattern was used as microsupercapacitor electrodes, and the electrochemical performance of the devices was evaluated.

## 2. Materials and Methods

### 2.1. Fabrication of PDMS Mold

**Figure 1:**
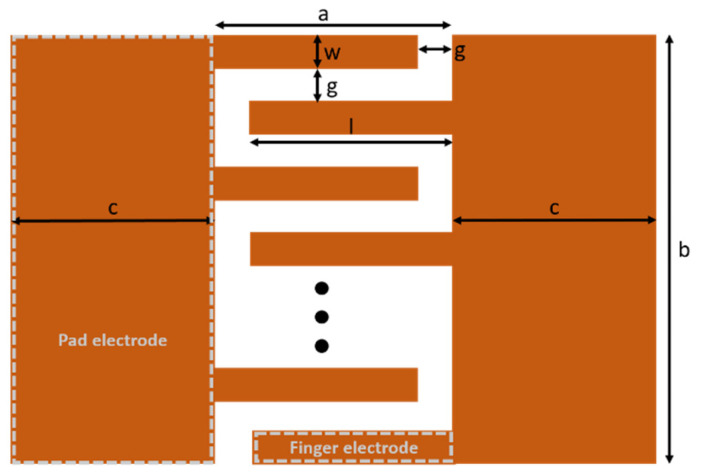
Schematic of the electrode pattern used for imprinting.

Figure 1 shows a schematic of the interdigitated electrode pattern used for furan imprinting. It consists of two pad electrodes and 10 parallel finger electrodes. Table 1 presents the dimensions of the imprinted pattern. The width of the finger pattern area (a) was 2.5 mm, and the width (w) and length (l) of each finger were 150 µm and 2.35 mm, respectively. The gap between the fingers (g) was set as 150 µm, and the length of the entire electrode (b) was 3 mm. The total active electrode area was 3.53 mm^2^ (2.35 mm × 0.15 mm × 10) and was used to calculate the specific capacitance.

To produce the same furan pattern as the designed electrode structure on the substrate by the imprinting process, a silicon master pattern identical to the electrode structure was fabricated on a silicon wafer by photolithography and reactive ion etching. The PDMS mold was prepared using a 10:1 w/w mixture of PDMS elastomer (Sylgard 184 A; Dow Corning Co. Ltd., Midland, MI, USA) and a curing agent (Sylgard 184 B; Dow Corning Co. Ltd., Midland, MI, USA). The PDMS mixture was poured on the silicon master with a positive structure and cured at 100 °C for 3 h to produce a PDMS mold with a negative structure.

### 2.2. Preparation of Imprinted Furan Pattern

In the proposed imprinting and laser pyrolysis process for fabricating carbon electrodes on a substrate, the adhesion between the imprinted pattern and the substrate needs to be improved. To evaluate the performance of the fabricated carbon electrode, the carbonized structure needs to be electrically separated from the underlying Si substrate. Thus, a SiO_2_ insulating layer was formed between the substrate and the furan pattern [33]. The SiO_2_ layer was formed on the silicon substrate by a thermal oxidation process using a tube furnace (modified MIR-TB600-H2, Mirfurnace Co. Ltd., Pocheon, Korea) at 1000 °C for 2 h. To improve the adhesion with the imprinted material, the -OH group of the SiO_2_ layer was activated by O_2_ plasma treatment [34].

In this study, furan resin (KC-5302 FA, Kangnam Chemical Inc., Seoul, Korea) was used as a precursor material to fabricate the porous carbon microelectrode. For the imprinting process, the curing agent (p-toluenesulfonic acid monohydrate; Kanto Chemical Co. Inc., Tokyo, Japan) was diluted in a small amount of ethanol (99%; Duksan Co. Ltd., Ansan, Korea) and mixed with the furan resin at a ratio of 3:97 by weight. The furan resin mixture was dropped on the substrate and carefully covered with the PDMS mold to avoid the formation of air bubble defects. Subsequently, it was thermally cured at up to 100 °C for 6 h to prepare the imprinted furan pattern. During the thermal curing process, a compression pressure of 16.6 kPa was applied to minimize the formation of a residual layer. The thickness of the furan pattern film was approximately 125 µm.

Although a compression pressure was applied during the imprinting process to minimize the formation of a residual layer, a thin residual layer formed between the electrode patterns. This thin residual layer can cause short circuiting between the finger electrodes after the carbonization process. Therefore, in this study the residual layer was selectively ablated using a nanosecond laser (Nano L200-20; wavelength: 532 nm; pulse duration: 6–9 ns; repetition rate: 20 Hz, Litron) before the CO_2_ laser pyrolysis process.

### 2.3. Laser Pyrolysis

The laser pyrolysis of furan polymer was performed using an economical CO_2_ laser engraver (C40–60, Coryart). The imprinted furan pattern was raster scanned with a continuous wave CO_2_ laser beam (1/e^2^ diameter: 125 µm). The CO_2_ laser power was determined by using a power meter (Nova II, Ophir) and a laser thermal power sensor (30(150)A-BB-18, Ophir). A rectangular scanning area was set so as to enclose the entire pattern, including the fingers and pad electrodes. The imprinted furan pattern was scanned along the lateral direction of the finger electrode at a scanning speed of 30 mm/s with a raster-scanning interval of 0.1 mm. Energy densities for 1, 2, and 3 W are converted to approximately 26.7, 53.3, and 80 J/cm^2^, respectively. Laser scanning was performed only once for each polymer sample, as repeated laser irradiation may result in different pyrolysis states.

### 2.4. Fabrication of Microsupercapacitor

A microsupercapacitor was fabricated using the carbonized interdigitated pattern as planar symmetric electrodes. A polyvinyl alcohol (PVA)-sulfuric acid (H_2_SO_4_) gel was used as the electrolyte. The electrolyte was prepared by mixing 3 g of PVA powder (molecular weight: 89,000–98,000, 341584, Sigma-Aldrich, St. Louis, MO, USA) and 3 mL of 1 M H_2_SO_4_ solution (ACS reagent grade, Sigma-Aldrich) with 30 mL of deionized water. This mixture was heated using a hotplate and stirred with a magnetic bar for 6 h at 90 °C, at the end of which the electrolyte turned transparent, indicating sufficient dissolution. A Cu tape was attached to the end of each pad electrode, which served as the current collector. The Cu tape was then sealed using Kapton tape to protect the Cu from the electrolyte. Finally, 20 μL of PVA-H_2_SO_4_ gel electrolyte was applied to the active finger electrode area. The electrochemical performance of the microsupercapacitor was evaluated using a potentiostat/galvanostat (Biologic, SP-150, Bio-Logic Science Instruments).

## 3. Results and Discussion

### 3.1. Laser-Induced Carbonization of Imprinted Furan Pattern

**Figure 2:**
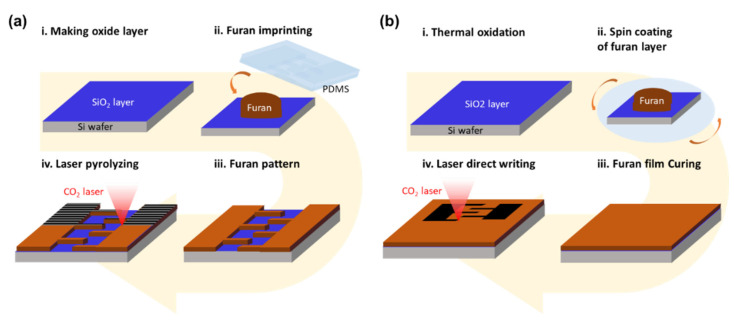
Schematic of furan imprinting and laser pyrolysis process (**a**) and conventional laser direct writing (**b**).

Figure 2a shows the schematic of the proposed CO_2_ laser pyrolysis process used to produce the carbon microelectrode. In the conventional laser direct writing of carbon on polymer (Figure 2b), a uniform continuous polymer film is scanned with a laser beam along a programmed path. Pyrolysis is localized near the scanned area, while the unexposed surface remains pristine. For example, Lin et al. patterned laser-induced graphene (LIG) by scanning a polyimide film using a CO_2_ laser and used the produced LIG micropattern as interdigitated microsupercapacitor electrodes [10]. However, the laser pyrolysis process proposed in this study is different from the above-mentioned method in that the carbonization is performed by the laser irradiation of a pre-patterned polymer structure. As described in Figure 2a, the pre-pattern was prepared by imprinting furan, and its surface was carbonized by laser beam sweeps. The SiO_2_ area was also exposed to the laser beam; however, it remained unaffected.

**Figure 3:**
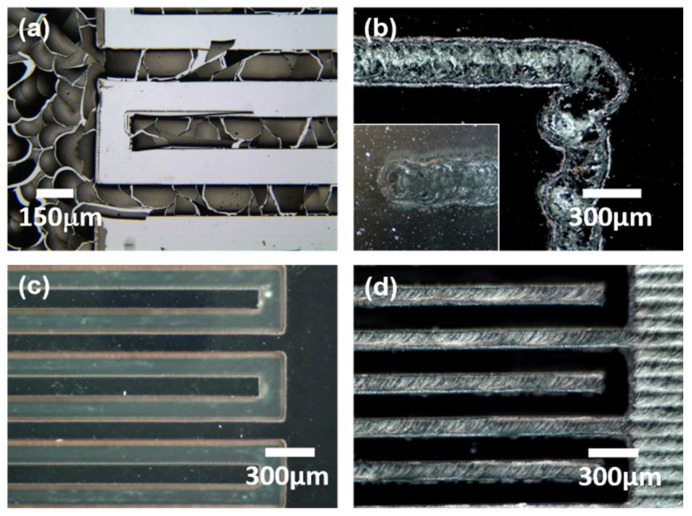
Top-view optical microscopy images of (**a**) fragmented furan pattern due to the high-temperature carbonization of the imprinted pattern, (**b**) laser-pyrolyzed pattern near a turning point of direct laser writing (inset: start point of laser scanning), (**c**) imprinted furan pattern, and (**d**) laser-pyrolyzed pattern on imprinted furan.

In addition to laser pyrolysis, the polymer pattern could be carbonized using an electric furnace [35,36]. As demonstrated in our earlier study, a vitreous carbon mold can be successfully produced from furan by high-temperature (1000 °C) pyrolysis using an electric furnace [37]. However, when the imprinted furan micropattern (30 µm thick for this sample) was heated at the same high temperature under a nitrogen gas flow, severe cracks were generated due to the shrinkage of the furan structure by pyrolysis [38]. As shown in Figure 3a, the furan layer delaminated from the substrate, possibly because of the weak adhesion between the carbonized film and the substrate.

When laser direct writing (power: 2.6 W or energy density: 69.3 J/cm^2^; scanning speed: 30 mm/s) was implemented on a spin-coated furan film (thickness: 30 µm) as described in Figure 2b, issues related to laser direct writing, as mentioned in the introduction, were observed; that is, circular regions appeared at the start/end points of the beam scanning path and at the corners where the scanning direction changed (Figure 3b). Moreover, deceleration/acceleration near these points could cause an abnormal temperature rise due to the prolonged exposure to the laser, resulting in serious thermal damage. In addition, when the length scale of the micropattern was comparable to the beam size, producing the carbonized pattern with dimensions precisely matching those of the original design became challenging. This is because the line width of laser pyrolysis changes depending on the laser power, which should be simultaneously tuned to obtain the desired pyrolysis depth and quality.

The laser carbonization method proposed in this study can overcome the limitations of the existing carbonization patterning techniques and obviates the necessity of preprogramming the laser writing path according to the electrode shape. The pre-patterned furan film was obtained by an imprinting method, and the entire rectangular area containing the electrode pattern was uniformly irradiated with the laser for carbonization. Figure 3c shows an optical microscopy (OM) image of the imprinted furan pattern before laser carbonization. Finger electrodes with uniform dimensions were successfully imprinted as designed. Figure 3d shows the OM image of the pattern carbonized with the laser. As can be seen, the shape of the carbonized electrode is consistent with that of the original imprinted pattern. This demonstrates the novelty of our approach in the precise production of laser-pyrolyzed carbon micropatterns.

### 3.2. Morphology of Laser-Pyrolyzed Carbon Structure

**Figure 4:**
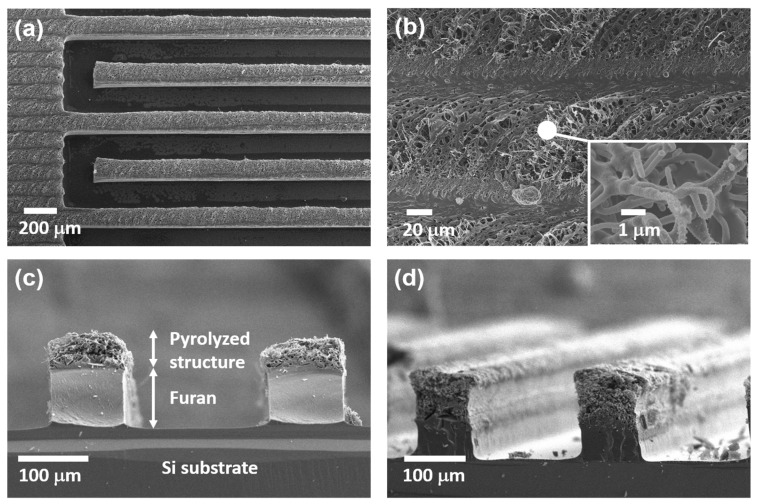
(**a**) and (**b**) Top-view SEM images of laser-pyrolyzed microsupercapacitor electrode (laser power: 2 W; scanning speed: 30 mm/s). The inset shows a high-magnification FE-SEM image of the carbonized structure. (**c**) Cross-sectional SEM image of partially pyrolyzed finger electrodes fabricated with a laser power of 1 W and scanning speed of 30 mm/s. (**d**) Inclined-view SEM image of pyrolyzed finger electrodes fabricated with a laser power of 2 W and scanning speed of 30 mm/s.

The carbonized structure produced by laser pyrolysis was analyzed by scanning electron microscopy (SEM). Figure 4a shows the top-view SEM image of the carbonized structure produced at a laser power of 2 W and a scanning speed of 30 mm/s. Even after laser pyrolysis, the carbonized pattern maintained the sharp edges of the original pattern. In addition, the carbonized structure was free of cracks and delamination. Figure 4b shows the SEM image of the carbonized surface of the pad electrode area. Numerous pores are present, which indicates that the material can be used for supercapacitor electrodes. The inset of Figure 4b shows a high-magnification field emission SEM (FE-SEM) image of the carbonized surface. As can be seen, the microstructure comprises nanoscale fibrous and particulate carbon materials.

Figure 4c,d show the cross-sectional SEM images of the finger electrode. The samples shown in Figure 4c,d were produced with a laser power of 1 and 2 W, respectively, and a scanning speed of 30 mm/s. The SEM images show that the laser carbonization proceeded to a limited depth because of a short optical absorption depth and, thus, limited heat penetration. The underlying unaffected furan connected the produced carbon with the substrate, whereas complete pyrolysis in a furnace caused delamination (Figure 3a).

### 3.3. Pyrolysis Depth Change with Variation in Laser Power

**Figure 5:**
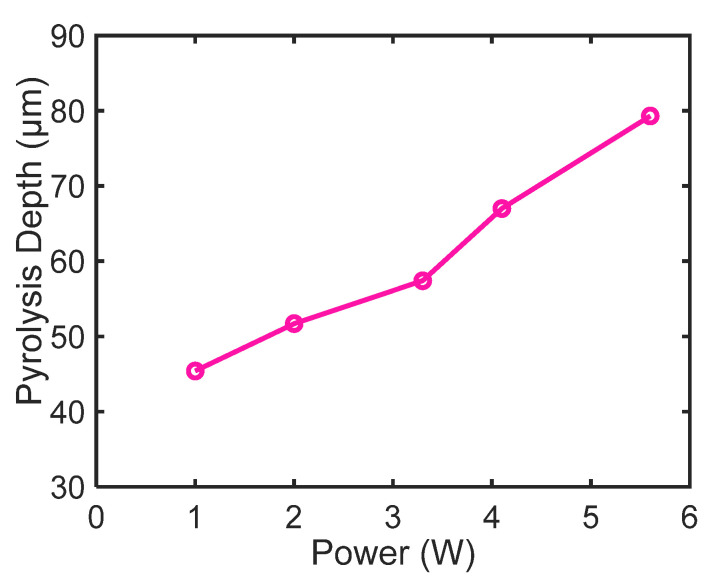
Variation in pyrolysis depth as a function of laser power (laser beam diameter: 125 μm).

To investigate the effect of laser power on carbonization depth, laser pyrolysis was performed by varying the CO_2_ laser power. To measure the depth of carbonization, the cross-section images were analyzed using an optical measuring microscope (STM-6, Olympus Co. Ltd., Tokyo, Japan), and the depth was confirmed by SEM. Figure 5 shows the variation in the pyrolysis depth of the pad electrode region with laser power. With an increase in laser power, the laser pyrolysis depth increased linearly from ~45 µm for 1 W power to ~80 µm for the highest power of 5.6 W (149 J/cm^2^). In contrast, in the finger electrode region, which consists of thin pattern lines, the carbonized electrode peeled off at laser powers higher than 3.3 W (88 J/cm^2^). Therefore, the laser power for the fabrication of microsupercapacitor electrodes by laser pyrolysis was limited to less than 3.3 W.

### 3.4. Raman Spectroscopic Analysis of Laser-Pyrolyzed Carbon

**Figure 6:**
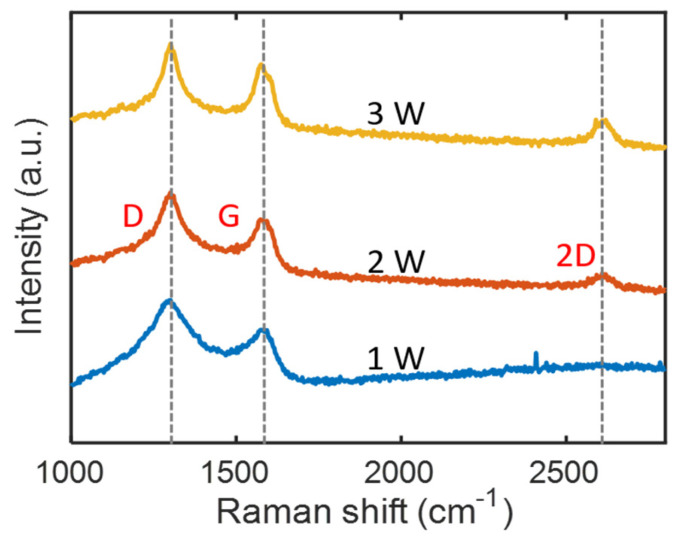
Raman spectra of laser-pyrolyzed microsupercapacitor electrodes produced at 1, 2, and 3 W.

To further determine the structural characteristics of laser-pyrolyzed carbon, Raman spectroscopy was performed for a series of samples produced at laser powers of 1, 2, and 3 W. The Raman spectra of graphitic carbon materials generally exhibit a D peak (1345 cm^−1^), G peak (1585 cm^−1^), and 2D peak (2700 cm^−1^). While the D peak arises from the presence of lattice defects in the crystal, the G peak originates from the stretching vibrations of the sp^2^ carbon lattice of the graphitic carbon structure. The Raman spectrum of few-layer graphene exhibits a pronounced 2D peak [39]. Figure 6 shows the Raman spectra of the sample surfaces. The difference in the D-to-G peak intensity ratios (I_D_/I_G_: ~1.3) of the samples fabricated at different laser powers is insignificant. However, with an increase in laser power from 1 to 3 W, the widths of the D and G peaks decreased, and the peaks became sharper. The 2D peak began to appear at a laser power of 2 W or higher, and the sample fabricated at 3 W exhibited a higher intensity 2D peak. As Lin et al. discussed in their study on laser-induced graphene, the 2D peak indicates the presence of randomly stacked graphene layers [10]. Thus, the increased 2D peak intensity suggests the production of graphene-like carbon at the increased laser power. Our observations on the Raman spectra are consistent with the result reported by Cai et al. [12]. They correlated the change in the Raman signal with the compositional change caused by increased laser power. They revealed that the carbon content decreased at a high power, whereas the oxygen content increased by oxidation.

### 3.5. Electrochemical Characteristics of Laser-Pyrolyzed Microsupercapacitor Electrode

**Figure 7:**
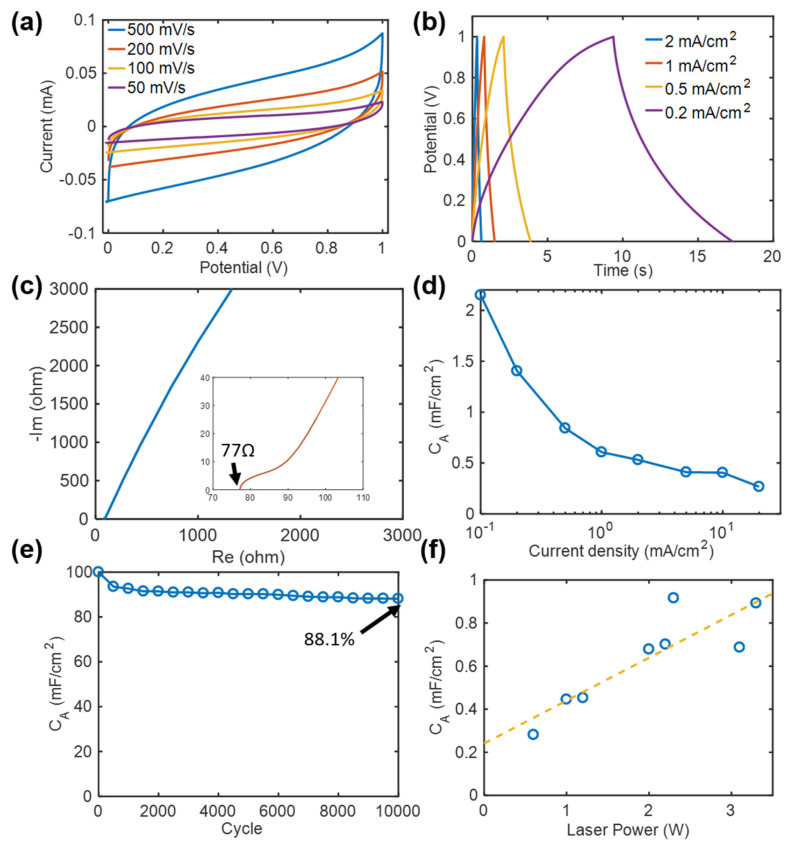
Electrochemical characteristics measured in a two-electrode configuration: (**a**) cyclic voltammetry (CV) curves of laser-pyrolyzed microsupercapacitor electrode (laser power: 2.2 W), (**b**) galvanostatic charge-discharge (CC) curves of laser-pyrolyzed microsupercapacitor electrode, (**c**) electrochemical impedance spectroscopy (EIS) curve of the microsupercapacitor (inset: EIS curve near the x-intercept), (**d**) C_A_ of the microsupercapacitor calculated from the CC curves at various current densities, (**e**) cyclic charging/discharging stability of the microsupercapacitor at 2 mA/cm^2^, and (**f**) C_A_ of microsupercapacitor electrodes fabricated at various laser powers.

Microsupercapacitors were fabricated using the carbonized pattern as interdigitated symmetric electrodes, and the electrochemical performance of the supercapacitor was evaluated using a potentiostat/galvanostat. Figure 7 shows the electrochemical testing results. Figure 7a–e show the electrochemical performance of the supercapacitor fabricated at a laser power of 2.2 W (58.7 J/cm^2^). Figure 7a shows the cyclic voltammetry (CV) loops measured at scan rates of 50, 100, 200, and 500 mV/s. At all the scan rates, the CV loops exhibit a pseudo-rectangular shape that is characteristic of carbon-based electric double layer capacitors (EDLCs). Figure 7b shows the constant current charge/discharge (CC) curves measured in the voltage range of 0–1 V at current densities of 0.2, 0.5, 1, and 2 mA/cm^2^. With an increase in the current density, the charging and discharging times decreased. Moreover, the CC curve shape resembles a triangle, which is characteristic of ideal EDLCs.

Figure 7c shows the electrochemical impedance spectroscopy (EIS) curve measured in the frequency range of 800 kHz to 100 mHz. The EIS curve shows a straight line with a slope, while an ideal EDLC exhibits a vertical shape in the low-frequency region. This indicates that the porous carbon structure formed by laser carbonization is relatively ineffective for ion transport via diffusion [40,41]. The inset of Figure 7c is an enlarged graph of the EIS curve showing its intersection with the *x*-axis, which reveals an ESR of approximately 77 Ω.

Figure 7d shows the specific capacitances (C_A_) in the current density range of 0.1–20 mA/cm^2^, which were calculated based on the galvanostatic charge-discharge (CC) data. The following equation was used to calculate C_A_ (mF/cm^2^):

C_A_ = (I∆t)/(A∆V), (1)

where I is the current (A), ∆t is the discharge time (s), A is the active electrode area including finger electrodes (cm^2^), and ∆V is the voltage window (V) in the constant current discharge curve excluding the IR drop. With an increase in the current density, the specific capacitance decreased due to the limited ion transfer rate.

Further, constant current charge and discharge cycling was performed for 10,000 cycles at a current density of 2 mA/cm^2^, and the retention rate of the capacitance was measured. The storage capacity in the first cycle was assumed to be 100%, and the change in storage capacity with cycling was evaluated. Figure 7e shows that the capacity retention rate gradually decreased with cycling; however, approximately 88.1% of capacitance was retained after 10,000 cycles, which indicates a relatively high capacity retention rate.

To investigate the effect of laser pyrolysis power on capacitance, a series of microsupercapacitors were fabricated by varying the laser power in the range of 0.6–3.3 W. At laser powers lower than 0.6 W (16 J/cm^2^), laser pyrolysis was unstable, and the capacitance was negligibly low or undetectable. At a laser power of 3.3 W (88 J/cm^2^) or higher, the irradiated finger electrode peeled off from the substrate due to excessive heating, and the microsupercapacitor could not be fabricated. The specific capacitance was calculated using the constant current charge/discharge data and Equation (1). Figure 7f shows the effect of laser power on capacitance. With an increase in laser power, the specific capacitance increased almost linearly. This is because a larger amount of porous carbon was generated at a higher pyrolysis temperature induced by the high laser power. In addition, more graphitic and electrically conductive carbon was generated at a higher pyrolysis temperature, as determined by Raman spectroscopic analysis (Figure 6). Consequently, the specific capacitance of the unit active electrode area increased. The maximum specific capacitance was ~0.92 mF/cm^2^ at 1 mA/cm^2^. Based on the CV data, the capacitance of the same supercapacitor was calculated to be ~1.84 mF/cm^2^ at 100 mV/s. This value is not as high as the capacitance of laser-induced graphene microsupercapacitors [8,10], but is comparable to or higher than those of other laser-pyrolyzed carbon microsupercapacitors [9,23,42].

## 4. Conclusions

This study proposes a new laser carbonization method to overcome the limitations of existing micro-carbonization techniques based on laser direct writing. The micropattern was pyrolyzed by irradiating with a CO_2_ laser after imprinting a furan resin on a silicon wafer. The fabricated microelectrode almost fully retained the imprinted shape. Thus, carbon electrodes that match the original design dimensions can be produced using this method. The fabricated carbon microelectrode had a porous structure, and could thus be used as a microsupercapacitor. The microsupercapacitor exhibited the typical characteristics of EDLCs. The specific capacitance of the microsupercapacitor increased with an increase in CO_2_ laser power. In this study, the applied laser power was limited due to the electrode peeling problem encountered at high temperatures; therefore, the power storage capacity was limited to approximately 0.92 mF/cm^2^. However, it is expected that the storage capacity can be further improved by using polymer materials other than furan for imprinting processes to produce high-quality pyrolyzed carbon in subsequent studies.

## Figures and Tables

**Table 1:** Dimensions of the electrode pattern.

Location	Dimension (µm)
Lateral length of active device area (a)	2500
Vertical length of active device area (b)	3000
Pad electrode width (c)	5000
Finger electrode width (w)	150
Finger electrode length (l)	2350
Gap between finger electrodes (g)	150
